# Advances in Intracranial Pressure Monitoring and Its Significance in Managing Traumatic Brain Injury

**DOI:** 10.3390/ijms161226146

**Published:** 2015-12-04

**Authors:** Usmah Kawoos, Richard M. McCarron, Charles R. Auker, Mikulas Chavko

**Affiliations:** Department of NeuroTrauma, Naval Medical Research Center, Silver Spring, MD 20910, USA; richard.m.mccarron.civ@mail.mil (R.M.M.); Charles.r.auker.ctr@mail.mil (C.R.A.); mikulas.chavko.ctr@mail.mil (M.C.)

**Keywords:** intracranial pressure, invasive methods, non-invasive methods, telemetry, waveform analysis, traumatic brain injury

## Abstract

Intracranial pressure (ICP) measurements are essential in evaluation and treatment of neurological disorders such as subarachnoid and intracerebral hemorrhage, ischemic stroke, hydrocephalus, meningitis/encephalitis, and traumatic brain injury (TBI). The techniques of ICP monitoring have evolved from invasive to non-invasive—with both limitations and advantages. Some limitations of the invasive methods include short-term monitoring, risk of infection, restricted mobility of the subject, *etc*. The invasiveness of a method limits the frequency of ICP evaluation in neurological conditions like hydrocephalus, thus hampering the long-term care of patients with compromised ICP. Thus, there has been substantial interest in developing noninvasive techniques for assessment of ICP. Several approaches were reported, although none seem to provide a complete solution due to inaccuracy. ICP measurements are fundamental for immediate care of TBI patients in the acute stages of severe TBI injury. In severe TBI, elevated ICP is associated with mortality or poor clinical outcome. ICP monitoring in conjunction with other neurological monitoring can aid in understanding the pathophysiology of brain damage. This review article presents: (a) the significance of ICP monitoring; (b) ICP monitoring methods (invasive and non-invasive); and (c) the role of ICP monitoring in the management of brain damage, especially TBI.

## 1. Introduction

**Figure 1:**
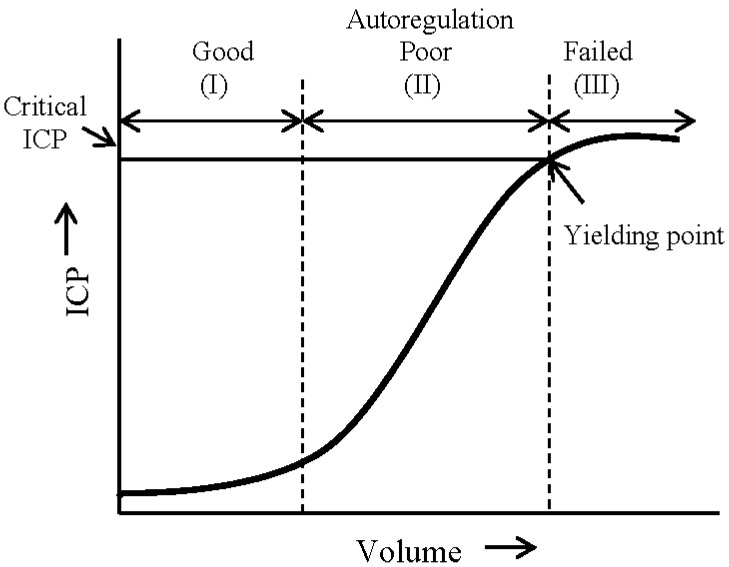
A pressure–volume curve representing the relation between cranial volume and ICP. The three sections on this curve show the regions of good (I), poor (II), and failed (III) compensatory mechanisms which are involved in autoregulation of cerebrovascular reactivity (modified from [3]).

Intracranial pressure (ICP) is a resultant of the pressure applied by the components within an inflexible and rigid skull. Changes in ICP are dependent on factors such as the expansion of intracranial volume, volume distribution of the components (brain, blood, cerebrospinal fluid (CSF), lesions, edema, *etc.*), elasticity of the components, and the presence of lesions. The Monro-Kellie doctrine states that the total amount of the intracranial volume of blood, brain, and CSF remains constant, and that an increase in any one of these must be compensated by an equivalent decrease in another [1,2]. A dynamic, non-linear relation exists between the intracranial volume and ICP. Figure 1 presents a conceptual intracranial pressure–volume curve [3].The pressure-volume relation can be presented in the form of a sigmoid curve with three distinct segments. In segment I, the compensatory reserves adequately prevent significant increase in ICP while the intracranial volume of one (or more) component(s) rises. In segment II, the compensatory reserves, in response to autoregulation processes, poorly combat against the increase in volume. In this segment ICP seems to have a near linear response to the increase in volume until a yielding point is reached. After a certain yield point (segment III), when autoregulation fails and the total intracranial volume cannot be maintained constant, ICP saturates near its critical value.

The thresholds of ICP are mostly defined by clinical experiences and the underlying condition(s) for pathological ICP [4]. There is no clearly defined common threshold of ICP that applies to all neurological disorders or brain trauma for warranting an immediate treatment. The common consensus for a normal range of ICP is 5–15 mm·Hg [4,5,6,7]. ICP levels of 15–25 mm·Hg may be considered tolerable, 25–40 mm·Hg is severely elevated, and levels over 40 mm·Hg are beyond the yield point and cannot be tolerated for extended duration of time [4]. The Brain Trauma Foundation guidelines recommend that treatment should be initiated when ICP threshold is greater than 20 mm·Hg for Level II trauma [8]. There is more to ICP monitoring than merely following the mean value of the pressure. Measurement of mean arterial pressure (MAP) and ICP is also used to estimate cerebral perfusion pressure (CPP). Thus ICP is not only monitored for its own management, but also for derivation of CPP using the formula: CPP = MAP − ICP. Another cerebrovascular value that can be derived from ICP is the pressure–reactivity index, which is used to evaluate cerebrovascular reaction by screening changes in ICP in response to slow spontaneous variations in arterial blood pressure (ABP) [3,9]. Under normal conditions, cerebral blood flow (and consequently ICP) is inversely proportional to changes in ABP. However, during the state of impaired cerebral autoregulation, an increase in ABP can lead to hyperemia, formation of vasogenic edema, and a secondary rise in ICP [3]. Cerebrovascular pressure reactivity is the capability of cerebral vessels to respond to changes in arterial pressure. The cerebrovascular pressure–reactivity index is calculated as the moving correlation coefficient between mean ICP and mean ABP [10]. Pressure–reactivity index is used to evaluate the cerebrovascular reactions by determining the response of ICP to slow spontaneous changes in ABP [11,12]. Advances in technology have enabled us to measure ICP waveform in addition to simply providing mean values of the pressure. Modern computational algorithms, capable of ICP waveform analysis can extract indicators of the dysfunction of cerebral vasoreactivity. A description of the components of ICP waveform and some computational methods is provided in Section 4 and Section 5.

### 1.1. Significance of ICP Monitoring

Any factors leading to an increase in the cranial volume could contribute to intracranial hypertension (ICHTN). An accurate knowledge of ICP can assist in managing patients with space occupying lesions, head injuries, pathological conditions causing an abnormal ICP; and following cranial surgeries. A significant reason for death and long-term disability due to head injuries and other intracranial pathological conditions is an elevation in the ICP. ICP monitoring has proven to be valuable, indeed often lifesaving, in the acute care of TBI [13], hydrocephalus [14], drowning [15] inflammatory and related cerebral diseases such as Reye’s syndrome [16], intracranial hemorrhage [17], cryptococcal meningitis [18], and postoperative sub-occipital brain tumors [19]. Most of these patients have headaches and other symptoms suggestive (but not always indicative) of raised ICP and continuous access to ICP levels may greatly facilitate their management.

In the recent past, ICP was considered to be an indicator of evolving underlying pathological processes resulting in high morbidity and mortality rate. Sustained ICP values of greater than 20–25 mm·Hg are considered pathologically significant due to a high correlation between elevated ICP and mortality [11]. At the same time it is inaccurate to believe that normal ICP levels may not be associated with neurological disorders. Often times metabolic and pathological processes are initiated before significant increases in ICP are seen [20]. A continuous and accurate measurement of ICP as part of the neurological armamentarium is very important in understanding disturbances in brain function.

TBI is a type of head injury that causes temporary or permanent alteration in brain function. The causes of TBI include automobile accidents, falls, sports injuries, assaults, wars, *etc*. Between 2006 and 2010 the rate of emergency department visits due to TBI had increased by 29% [21]. TBI is also a leading cause of neurocognitive impairment in the military population [22,23]. Expert guidelines for the use of ICP and CPP have been developed for the management of TBI [8]. Refinements in ICP monitoring technology have contributed to its contemporary extensive usage in managing brain trauma. The application of ICP monitoring in TBI is described in Section 5.

### 1.2. ICP Technology

ICP monitoring by ventricular catheters was described in some early reports by Guillaume and Janny in 1951 [24], and Lundberg in 1960 [25]. The early measurements were done by using U-tube manometry in which CSF was allowed to flow in the tube until the flow was stopped by backpressure. This backpressure was used to estimate ICP. An electronic transducer was used to measure ventricular ICP in patients with various intracranial pathologies [24]. The use of strain-gauge transducers used for ICP measurements improved the accuracy of measurements and marginally increased the practicality of such methods. These transducers were initially connected to patients with a fluid (saline) filled catheter under stringent test conditions. Patient mobility was restricted and the measurements were affected by posture [25,26,27]. A number of tethered systems (fluid-filled catheters, transducer-tipped catheters [28,29], fiber optic systems [30,31,32], *etc.*) were investigated and some are being used in clinical practice at present [33,34]. Some of the disadvantages of catheter based and tethered systems are restricted patient mobility, risk of infection, sensor displacement, sensor dislodgement, inaccurate positioning, *etc*. Additionally, these systems may also encounter zero drift, lack robustness [28,35,36,37], and they cannot be recalibrated *in vivo*. Transducer-tipped intraventricular or intraparenchymal Camino sensors (Camino Laboratories, San Diego, CA, USA) have shown an average drift of up to 3.2 mm·Hg per day [36], and in 10% to 25% of cases the system failed due to technical complications [35,37,38]. An average bias of at least 5 mm·Hg in 24% of comparisons done between strain-gauge intraparenchymal Codman sensors (Johnson & Johnson Professional, Inc., Raynham, MA, USA), and the Camino system was also reported [28].

There is a conspicuous need for acute as well as long-term ICP monitoring systems, as several chronic diseases are associated with ICHTN. An implantable, wireless method for long-term monitoring of ICP is vital in managing patients suffering from brain trauma or disorders. Accurate and reliable ICP monitoring after neurosurgery is a basic requirement for ensuring an adequate treatment [39,40,41,42,43,44]. Direct measurements of ICP require neurosurgical intervention, which have risks associated with them. An implant placed during neurosurgery would be a useful adjunct to post-surgical patient care.

Direct methods (catheters and implants) of ICP monitoring are invasive in nature with potential serious complications associated with them. These methods may not be preferred unless a surgical intervention is otherwise required. The quest for indirect/non-invasive method that is accurate, safe and easy to use, and does not cause patient discomfort is ongoing. A detailed description of direct and indirect methods, their uses, advantages and disadvantages is presented in Section 4.

## 2. Key Factors to Be Considered in ICP Monitoring Technology

The optimal ICP monitoring method should be accurate, reliable, cost-effective, and cause minimal patient morbidity. Many factors are to be considered while designing and testing ICP monitors, which include accuracy, optimal positioning of the sensor, pressure gradients across brain, potential complications, safety, *etc*. The American National Standards Institute (ANSI)/Association for the Advancement of Medical Instrumentation (AAMI) have developed the standards for ICP monitoring devices [45], some of which are described here.

### 2.1. Device Accuracy

As per the ANSI/AAMI standards, ICP monitoring devices should have a reliable functional pressure range of 0–100 mm·Hg; provide an accuracy of ±2 mm·Hg in the range of 0–20 mm·Hg; and the maximum error should not exceed ±10% in the range of 20–100 mm·Hg. Invasive ICP monitoring allows pressure transduction by transducer-tipped catheters placed in the intracranial space or external strain-gauge transducers which are coupled to the intracranial space via fluid filled catheters. The transducer-tipped catheters are calibrated before their insertion in the intracranial space. These catheters cannot be recalibrated without the use of additional accessories, which increase the risk of complications. External strain-gauge transducers are easy to recalibrate, but obstruction in the fluid lines can be a source of inaccuracy. The external pressure transducer must be maintained at a constant level in reference to the patient’s head.

### 2.2. Transducer Position for ICP Measurements

ICP probes can be of intraventricular, intraparenchymal, subarachnoid, sub-dural, or epidural type. The optimal positioning of the transducer is determined by factors including the desired accuracy, duration of monitoring, acceptable drift and complications, therapeutic benefits, *etc*. The gold standard for transducer placement is the intraventricular site [46,47,48,49,50,51], which can also be therapeutically beneficial if a CSF drainage mechanism is included in the system. Intraparenchymal and sub-dural systems are comparable to intraventricular methods [51,52,53]; however, some studies have reported that these methods do not always correlate well with the gold standard [47,54,55,56]. Fluid filled epidural or sub-arachnoid [48,49,50,57,58,59] and pneumatic epidural methods [60,61,62] are not preferred techniques as they are less accurate in reference to the intraventricular methods.

### 2.3. Pressure Gradients

In determining the ideal site for pressure transduction it is noteworthy to mention that small pressure gradients exist within the central nervous system, which may be altered by pathology or trauma [63,64]. These intercompartmental pressure gradients may not vary significantly under normal physiological conditions [65,66]. It is very important to determine the optimal location for introducing a pressure transducer or catheter where local pressure gradients may not influence the accuracy of ICP measurements especially while treating conditions like hydrocephalus, idiopathic ICHTN, sub-arachnoid hemorrhage, *etc*. There are no conclusive studies quantifying these pressure gradients and describing the different conditions under which such gradients may have a significant impact on ICP. However, in trauma conditions, a localized increase in ICP is very likely and should be treated with urgency especially when poor correlation is observed between ICP and clinical symptoms [67].

### 2.4. Complications

The complications that arise during ICP monitoring include infection, hemorrhage, dislodgment of the sensor, malfunction, drift, and inaccurate positioning. These complications can potentially produce inaccurate ICP readings, reduce the efficacy of the procedure, and cause further damage to already injured brain.

## 3. Methods of ICP Monitoring

ICP measurement and analysis can be achieved by direct, indirect or analytical methods. Direct or invasive monitoring provides direct signals from a CSF occupying space, brain tissue or dura mater. Some examples of direct methods are ventricular, sub-dural, and epidural pressure measurements. The indirect methods are non-invasive in nature, which are based on the derivation of ICP from other parameters with a known relationship with ICP. The measuring of optic nerve sheath diameter by transocular ultrasound, tympanic membrane displacement (TMD) by cochlear fluid pressure (CCFP) analyzer, or tissue oxygenation by near infrared spectroscopy (NIRS) are some non-invasive methods for inferring ICP. Beyond ICP, dedicated computational models are used to calculate variables defining the pathophysiology. Some examples are CPP, pressure–reactivity index, brain compensatory reserve, impedance, and compartmental compliances.

### 3.1. Invasive

Invasive ICP monitoring can broadly be classified into three groups: fluid-filled systems, transducer-tipped catheters, and telemetric methods. Under certain circumstances with non-obstructed pathways of CSF flow, ICP may also be measured by lumbar puncture [68,69,70].

#### 3.1.1. Fluid-Filled Systems

These systems are comprised of a transducer connected to a fluid line which communicates with an intracranial compartment. The transducers are placed externally at a fixed level in reference to the patient’s head (at the level of tragus). Intraventricular catheters are the most accurate systems for ICP measurement [3,71,72]. The catheter is inserted in one of the lateral ventricles and the external transducer is held at the same level as the foramen of Monro. These systems are also used for ventricular drainage of CSF and administration of therapeutic agents. These systems are highly prone to infections, can be difficult to place, may cause brain trauma and progressive edema, and are likely to leak or get blocked. Sub-arachnoid screws, bolts or catheters in which the fluid line is placed in the sub-arachnoid space, are simpler than the intraventricular type. The rates of infection and brain trauma are low, but they have a tendency to underestimate ICP [49,67,73,74]. Spiegelberg catheters are specialized systems that use air for pressure transduction, can be automatically corrected for zero drift *in vivo*, and can also measure brain compliance [75,76,77].

#### 3.1.2. Implantable Transducer Catheter Systems

These systems do not use a fluid interface and the transducer is directly placed in the intracranial space. The transducers are of solid state (strain-gauge, piezoresistive, capacitive, *etc.*) [78,79], microchip [8,46,73] or fiber optic type [36,73]. Fiber optic transducers measure pressure based on the pressure-sensitive deflection of a flexible diaphragm. They transmit pressure dependent light through a fiber optic cable towards a displaceable mirror. The displacement of the mirror changes the reflection properties of the light that is used to determine ICP. The Integra Camino Advanced Monitor (CAM01, Integra LifeSciences, Plainsboro, NJ, USA) is a fiber optic system that measures ICP and brain temperature. Strain-gauge systems rely on the change in resistance in response to ICP, which is translated into a pressure dependent electronic signal. Some examples of strain-gauge systems are Codman MicroSensor (Codman and Shurtleff, Inc., Raynham, MA, USA), Raumedic Neurovent-P and S type (Raumedic AG, Helmbrechts, Germany) and Pressio (Sophysa, Orsay, France). Transducer-tipped catheters can measure intraventricular, intraparenchymal, sub-arachnoid or epidural ICP. In terms of accuracy, various ICP systems can be listed in the following order: intraventricular, intraparenchymal, sub-arachnoid, and epidural. Epidural systems have been shown to overestimate ICP [80], especially at higher pressure values [81].

Integrated catheter systems for simultaneous multi-parametric measurements are becoming a part of advanced neuromonitoring in intensive care units. An example of an integrated catheter system capable of simultaneously measuring ICP (strain-gauge sensor), temperature (thermocouple), and tissue oxygenation is Neurovent-PTO (Raumedic). A single catheter capable of multi-modality monitoring by merging laser Doppler and sensors for ICP, tissue oxygenation, and temperature was described and tested in porcine models by Leidorf *et al.* [82].

#### 3.1.3. Telemetric Systems

ICP monitoring done via catheter systems can only be done in hospitals and clinical settings under controlled conditions to minimize the risks associated with such methods. For long-term ICP monitoring tethered systems are not desirable due to limited patient mobility, in addition to other risk factors. A number of methods have been developed to measure ICP by a completely implantable device with data transmission via telemetry [78,81,83,84,85,86]; however, their application is mostly restricted to basic non-clinical research. Epidural, sub-arachnoid, and intraventricular telemetric systems and their use in monitoring ICP in hydrocephalus and TBI in animals were described [78,81]. Neurovent P-tel and S-tel (Raumedic AG, Helmbrechts, Germany) were evaluated to determine their reliability for long-term intraparenchymal and subdural ICP measurements conducted over 10 months [83]. All of intraparenchymal devices remained functional whereas 33% of the subdural devices lost their functionality. Both P- and S-type showed minor zero drift, but measured dynamic ICP signal with excellent stability. A telemetric device based on capacitive pressure sensing was described by Kroin *et al.*, for long-term monitoring of ICP [84]. This device was tested in dogs, where its function was compared with ICP measurements taken from a fluid-filled system with its catheter placed in the cisterna magna. The response of both methods to stimuli (compressing jugular vein, changing head elevation, and altering volume in cisterna magna) was evaluated and compared. It was demonstrated that for months the telemetric device produced acceptable drift when compared with the pressure measured from cisterna magna. Silasi *et al.*, used telemetry (PA-C10, Data Sciences Int., New Brighton, MN, USA) to measure epidural ICP for several days in freely moving rats [85]. The changes in ICP in response to Valsalva Maneuver were elicited as expected in anesthetized as well as awake rats. A fully implantable pressure-balanced telemetric system for short-term and long-term ICP measurements was reported to be safe, effective, reliable, and easy to use after conducting preliminary clinical studies [86].

### 3.2. Non-Invasive

Invasive methods of ICP monitoring are accurate and by far the best way to measure ICP in acute conditions. However, these methods are not desirable for sustained long-term monitoring due to the risk of infection, hemorrhage, pain, discomfort, *etc*. The idea of non-invasive ICP monitoring is attractive as it eliminates most problems associated with invasive monitoring. Some widely familiar non-invasive methods are described here.

#### 3.2.1. Impedance Mismatch

ICP can be detected from the impedance mismatch between carotid arteries and cerebral vessels as described by Swoboda *et al.* [87]. Their device consists of a pressure transducer, analyzer, and display and can be used as a bed-side monitor for measuring ICP non-invasively. The algorithm they developed extracts features (e.g., the time delay between systolic maximum and dicrotic notch), which have a known correlation with ICP.

#### 3.2.2. Tympanic Membrane Displacement (TMD)

The intracranial fluid system and the labyrinth are interconnected via the cochlear aqueduct; consequently, changes in the ICP have an effect on the mechanics of the audio-vestibular system. The displacement of the tympanic membrane in response to stimulation of the stapedial reflex is related to ICP [88]. CCFP is related to ICP and can be used as a non-invasive indicator of ICP [89,90,91]. A non-invasive TMD CCFP analyzer can assess the state of ICHTN. The TMD-CCFP analyzer was evaluated in laboratory and clinical settings. Wunderlich *et al.*, found a significant correlation between direct and TMD ICP measurements in cats [92]. The reliability of this technique is dependent on the tympanic membrane being intact and perilymphatic duct being passable, which imposes limitations on its applicability.

#### 3.2.3. Transcranial Doppler (TCD)

TCD ultrasonography relies on measurement of the velocity of blood flow in the middle cerebral artery. The ratio of the difference in systolic and diastolic flow velocity to mean flow velocity is described as the pulsatility index (PI). Various studies have reported correlation coefficients between PI and ICP which range from poor (0.22 [93]) to good (0.938 [94]). In a prospective study of 125 patients with severe head injury TCD was used to calculate PI, and ICP was measured clinically within the first 24 h of hospital admission [95]. A statistically significant correlation was found between PI and ICP (*r*^2^ = 0.6, *p* = 0.0001) and it was concluded that TCD is a valid method for predicting the outcome after 6 months of severe head injury. Voulgaris *et al.* reported that PI measurements permit an early identification of TBI patients with low CPP and high risk of cerebral ischemia [96]. They suggested that *in situ* ations where ICP monitoring is not feasible TCD can be used alone. On the contrary, Behrens *et al.*, reported that TCD PI is not an accurate method to determine ICP [93]. In a set of 35 patients, poor correlation was seen between ICP (measured intraparenchymally) and PI estimated using TCD (*r* = 0.22, *p* < 0.01). Thus the efficacy of TCD in estimating ICP remains controversial.

#### 3.2.4. Near Infrared Spectroscopy (NIRS)

NIRS is used to derive the levels of oxygen saturation by estimating deoxygenated and oxygenated hemoglobin in the brain. Some studies have demonstrated a relationship between slow vasogenic ICP waves and NIRS variables [97,98]. Weerakkody *et al.*, studied the synchronization between ICP and NIRS variables induced by vasogenic waves of ICP [97]. The increase in ICP was associated with the rise in the power of ICP slow waves. Slow variations in brain oxygen saturation coincide with the initiation of ICP slow waves. The fluctuations increase when the cerebral compensatory reserve is exhausted. Diedler *et al.*, have reported limitations of the NIRS technique in assessing cerebrovascular reactivity [99]. They suggested that the NIRS-based cerebrovascular reactivity index can be used as a noninvasive substitute for pressure–reactivity index, but only during phases with sufficient ICP slow wave power.

#### 3.2.5. Optic Nerve Sheath Diameter (ONSD)

The sub-arachnoid space between the dura and the white matter of the optic nerve communicates with the sub-arachnoid space of the brain. A rise in ICP leads to an expansion of the sub-arachnoid space surrounding the nerve which causes an increase in its diameter. The changes in ONSD can be estimated using transocular ultrasound. Tayal *et al.*, conducted a study on 59 adult head injury patients to determine if elevated ICP, as found on Computed Tomography (CT), can be accurately predicted by the changes in ONSD [100]. The ONSD method was found to have a sensitivity of 84% (95% CI 60% to 97%) and specificity of 73% (95% CI 59% to 86%) in detecting any traumatic head injuries that were found by CT. They concluded that this technique has the potential to be a sensitive screening test for raised ICP after adult head injury. Geeraerts *et al.* found a significant relationship between ONSD and invasively measured intraparenchymal ICP in sedated neurocritical patients [101]. They also reported a strong correlation between the changes in ICP and the changes in ONSD. For ICP > 20 mm·Hg, enlarged ONSD was a strong predictor of elevated ICP. In severe TBI patients a significant difference was found between ONSDs of patients with high ICP (when ICP is higher than 20 mm·Hg for more than 30 min in the first 48 h after admission) and normal ICP [102]. Other studies have reported ONSD ultrasonography to be an inadequate replacement for invasive ICP measurements [18,103].

#### 3.2.6. Fontanometery

In infants ICP can be estimated by pneumatic applanation fontanometer [104,105]. The device consists of tambour placed on the anterior fontanelle. As the externally applied pressure in tambour increases, the amplitude of pulsation increases to its maximum before it starts declining. The peak pulsation amplitude occurs when the tambour pressure equals the pressure inside the skull. Rochefort *et al.* used fontanometry to estimate ICP in four babies for seven days and found good correlation between estimated and measured ICP [106]. A fontanometer based on strain-gauge principle was found to produce accurate ICP estimation in hydrocephalic babies and neonates [107].

#### 3.2.7. Pulsed Phase Lock Loop (PPLL) Technique

The PPLL system generates a 500 kHz ultrasonic signal, which is transmitted through the temporal bone. The transmitted signal travels through the brain, is reflected by the inner surface of the contralateral side of the skull. A single transducer is used for transmitting and receiving the signal. A change in the diameter of the skull produces a phase shift, which causes a change in PPLL output voltage in proportion to cranial diameter pulsations [108].

ICP monitoring techniques are diverse and several factors (accuracy, invasiveness, cost, *etc.*) need to be considered before choosing a particular method. None of the non-invasive methods mentioned here are accepted for routine use in critical care settings.

## 4. ICP Waveform Analysis

The interpretation of ICP using a single number, either at one moment in time or as the average over a period of time (e.g., daily ICP), although useful, can often be misleading. A high-resolution view of ICP waveforms can be used for more sophisticated analysis of ICP. The signal artifacts due to electrostatic discharge and interference between equipment cannot be determined when ICP is viewed as a mean number. Electrostatic discharge can lead to an abrupt spike or a gradual drift in ICP; these can be detected and quantified by analyzing the mean wave amplitude of ICP pulse [67,109].

### 4.1. Waveform Analysis

**Figure 2:**
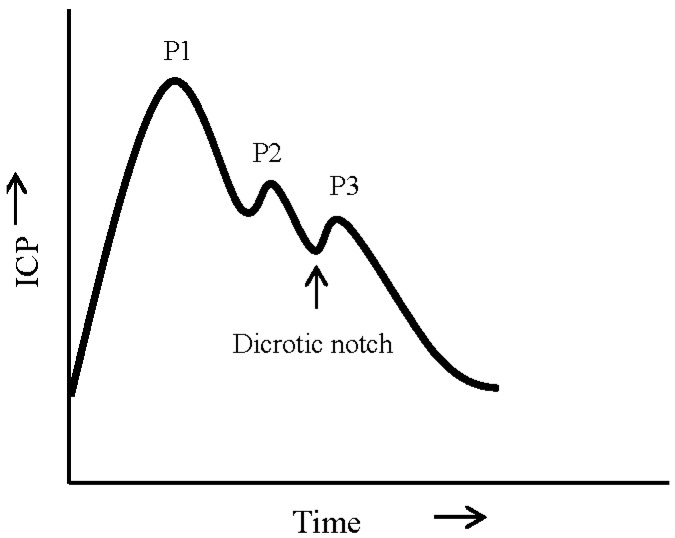
A single ICP pulse showing the components of ICP waveform. P1, percussion wave; P2, tidal wave; P3, dicrotic wave; and the beginning of P3 which aligns with the position of dicrotic notch on the arterial pulse.

A single ICP pulse is often characterized by three notches, progressively decreasing in amplitude, reflecting the propagation of arterial pulse pressure. Figure 2 is an example of an ICP pulse [4,67], showing P1, P2, and P3 notches, which indicate changes in ICP associated with the propagation of arterial pulse pressure. P1, P2, P3 notches correspond to the systolic or percussion wave, tidal wave, and dicrotic wave, respectively [4]. The shape of the ICP waveform is related to the mean ICP [110]. At low ICP, P1 is a very distinct peak. As mean ICP amplitude increases, the rate of increase in P2 is much higher than that of P1. The reversal of amplitudes of P1 and P2 peaks (P2 > P1) is an indication of exhausted cerebrospinal compensatory reserve. During cerebral dysfunction ICP waves may also be characterized by Lundberg A and B waves [25]. “A” or plateau waves are rapid fluctuations in pressure (50–100 mm·Hg) lasting for 5–20 min. Plateau waves are irregular in nature and indicate an acute compromise of autoregulation. “B” waves (0.5–2/min) could be an indicator of impaired cerebral function or may also appear naturally during different sleep stages [111]. Fluctuations in ICP due to respiration are minimal and may not be observable under normal conditions. Specific changes in ICP waveform can be used to measure progression of cerebral dysfunction.

### 4.2. Histogram Analysis

ICP histograms are produced by grouping ICP measurements into classes defined by standard intervals of pressure [4]. These histograms have been used to study dyssymmetry and flattening coefficients, and Gaussian distribution of ICP data [112]. The presence of plateau waves in ICP can possibly cause the histogram pattern to deviate from the underlying Gaussian distribution [25].

### 4.3. Frequency Analysis

An alternative method to analyze ICP waveform is the extraction of its three frequency components, which are: slow waves, respiratory waves, and pulse waves. Slow waves are found in the spectra of 0.05–0.0055 Hz. Respiratory waves correspond to the frequency of respiration. The pulse waves have harmonics representing P1, P2, and P3 notches. The frequency of the fundamental harmonic represents the heart rate [11].

## 5. Role of ICP Monitoring in Traumatic Brain Injury

ICP monitoring has become a cornerstone of care in managing TBI. In the United States the numbers of TBI cases that were being managed with ICP monitoring increased from 32% to 78% in 10 years (1995–2005) [8]. In critically injured patients, it is essential to rapidly assess the injuries and institute life-saving treatment protocols in a timely manner [113]. As per Brain Trauma Foundation guidelines [8], ICP should be monitored in Level II and Level III TBI. Level II consists of all salvageable TBI patients with Glasgow Coma Scale scores of 3–8 and an abnormal CT scan. Level III consists of severe TBI with normal CT scan with at least two of the following features: age > 40 years; unilateral or bilateral motor posturing; and systolic blood pressure <90 mm·Hg. The expert consensus in these guidelines highlights the monitoring and managing of ICP and CPP for the management of TBI. Measurement of ICP and MAP is used in derivation of CPP and designing targeted therapy during intensive care of acute brain injury patients. TBI results in the activation of primary and secondary pathophysiological processes which have an effect on ICP. In severe cases of TBI, elevated ICP is associated with mortality or poor clinical outcome. The main goal of ICP monitoring in TBI is to allow early detection of secondary damage and initiate immediate therapeutic intervention. The long-term continuous ICP monitoring is even beneficial as the pattern of changes in ICP can be used as a guide for individualized treatment. ICP monitoring in conjunction with other neurological monitoring can help in understanding the pathophysiological processes of the damage.

A study to evaluate the effect of intensity and duration of episodes of elevated ICP on the six-month neurological outcome from TBI reported a relationship between increased ICP and clinical outcomes [114]. This study analyzed ICP and MAP of 261 adults and 99 pediatric TBI cases, and modeled the relationship between the intensity and duration of ICP and the outcome. The relation between elongated periods of high ICP and worse outcome is nearly exponential in the adult and pediatric populations; however, the thresholds are different. ICP greater than 20 mm·Hg lasting more than 37 min in adults and 8 min in children were associated with the worst outcomes. When autoregulation is impaired, the tolerance for elevated ICP-duration burden is also reduced. Poor outcome in terms of survival rate and long-term functionality was found to be associated with ICP after TBI [115]. In [115], a randomized trial of 499 TBI patients, average ICP in the first 48 h of monitoring proved to be an independent predictor of mortality after moderate to severe TBI. Additionally, ICP was also a predictor of a composite outcome of functional and neuropsychological status after six months. However, there was no correlation between ICP and neuropsychological factors only.

In managing TBI continuous recording of ICP is a key neuromonitoring parameter. ICP is expressed in two ways, as “mean” and “grand mean”. The mean ICP is calculated by averaging stable ICP for at least 30 min and the grand mean ICP is derived from ICP recording acquired overnight during natural sleep cycles [11]. Some commonly observed patterns after TBI are presented as follows [11]. Low and stable ICP (<20 mm·Hg) is seen in mild to moderate head injuries. This pattern is also seen right after TBI, before the onset of brain edema. High and stable values (>20 mm·Hg) are most commonly seen after TBI. A and B waves are present in both time and frequency domains of ICP in severe TBI. Refractory ICHTN generally leads to a negative outcome unless surgical interventions are applied to relieve hypertension. ICP is also used to determine an index of pressure–volume compensatory reserve (RAP) which is the correlation coefficient between the fundamental amplitude of ICP and the mean ICP [11]. The average values of RAP and pressure–reactivity index are strong predictors of mortality after TBI. Additionally the power of slow waves should also be considered when predicting the outcome after TBI. Balestreri *et al.* [116] conducted a retrospective study on 96 head injury patients, out of which 57 died and 39 survived. It was concluded that an increase in ICP can be observed after head injury in both favorable and fatal outcomes. However, the indices derived from ICP waveform analysis are a significant tool in predicting the true outcome. The most significant difference between the two outcomes was found in the magnitude of slow waves, which were higher in patients with favorable outcome. In the patients with fatal outcome, ICP was significantly higher and pressure–reactivity index was severely compromised.

Increased ICP is strongly associated with unfavorable outcome and excessive CPP may diminish the likelihood of a favorable outcome following head trauma [115,117,118,119,120,121]. ICP thresholds are even lower for elderly and female populations [121]. Although the existing guidelines recommend ICP monitoring for managing TBI, some studies argue its benefits are limited [122,123]. Chesnut *et al.* reported that in patients with severe TBI, treatment focused on maintaining ICP below 20 mm·Hg was not shown to warrant superior care as compared to the one based solely on imaging and clinical examination [124]. In a review conducting meta-analysis of ICP monitoring studies, the reviewers reported a lack of sufficient evidence from clinical data in support of significantly superior outcome (*i.e.*, in terms of mortality after TBI) when ICP monitoring was included in the treatment protocol [122]. In an observational study, the utilization of ICP monitoring was associated with reduced mortality. However, variability in the frequency of ICP monitoring contributed modestly to variability in institutional mortality rates [123].

The discrepancies reported on the role of ICP monitoring in TBI are likely associated with methodological definitions and differences rather than the significance of ICP and its management. Some of the possible sources of discrepancies are: lack of details in the study design to adjust for injury severity; lack of standardization in ICP monitoring; focus on mortality outcomes; irregularities in study population selection; definition of ICP thresholds; management variability, *etc*. Basic neuromonitoring involving neurological examination, ICP and CPP measurements, CT scans with the addition of extended neuromonitoring (jugular venous oxygen saturation, partial tissue oxygen pressure, electrocorticography to determine cortical spreading depression, *etc.*) helps in the overall improvement of treatment options by characterizing functional influences, individualizing threshold values, and performing adaptive therapeutic intervention [125]. Multimodality extended neuromonitoring may be an efficient approach to understand individualized ICP/CPP thresholds and targets. A dynamic patient-tailored ICP/CPP target, based on autoregulation capacity of the cerebral vasculature is a preferable approach than the use of a general threshold for all TBI patients. It was suggested that ICP monitoring should be considered as an essential factor in the interpretation of information obtained from other intracranial monitors [126]. The recommendations for ICP monitoring in comatose TBI patients, as per the common consensus arrived by expert neurosurgeons and intensivists [127], are: (i) may not be required with normal initial CT scan; (ii) is recommended with early CT scans showing minimal signs of injury if the initial findings worsen over time; and (iii) should be performed if initial CT scan demonstrates diffuse injury with signs of brain swelling. Despite some controversies, ICP monitoring remains important in guiding treatment particularly after severe TBI, and is accepted as a standard of care in developed countries [128,129]. ICP management has the potential to influence clinical outcome, especially with supplemental data from monitoring like clinical examination and imaging, in particular when targeted and individualized treatment is provided [129].

Another parameter that has been of much interest in the management of TBI is CPP. Cerebral blood flow is dependent on CPP, which has an important role in maintaining adequate cerebral perfusion. CPP can be modified by altering ICP or MAP, but the optimal level of CPP for TBI likely depends on patient, time, and pathology of TBI [130]. Based on the common consensus, the Brain Trauma Foundation guidelines suggest that CPP should be maintained in the range of 50–70 mm·Hg [8]. A CPP management strategy is advocated by Rosner *et al.* that is based on the concept of vasodilatory cascade [131,132]. Under normal pressure autoregulatory response, a reduction in CPP stimulates vasodilation in order to maintain adequate cerebral blood flow. Based on the concept of vasodilatory cascade, increase in cerebral volume further reduces CPP by increasing ICP, thus setting up a cascade leading to ever reducing CPP. This cascade can be interrupted by increasing MAP which seems to reduce ICP [133]. Rosner *et al.* recommend maintaining CPP at greater than 70 mm·Hg in head injury patients to minimize cerebral ischemia. However, TBI often causes impairment of autoregulatory responses, which can lead to an increase in ABP causing higher cerebral blood flow, edema, and ICHTN. The “Lund approach” focuses on lowering ICP by reducing intracranial volumes [134]. This approach proposes that by reducing CPP there is a lower probability of vasogenic edema which in turn prevents the buildup of ICHTN. There is no well-established evidence that maintaining low or excessive CPP in TBI significantly impacts outcomes. Excessive CPP was associated with poor outcome after TBI due to acute respiratory distress syndrome [135]. A study of 427 patients prospectively studied in the multicenter, randomized controlled trial of the N-methyl-D-aspartate antagonist, Selfotel found that the most powerful predictor of neurological deterioration was ICP and there was no correlation of outcome with CPP as long as it was maintained greater than 60 mm·Hg [136]. Miller *et al.* suggested that ICP targeted therapy for TBI has more success when the treatment focuses on the underlying pathophysiology *i.e.*, vascular or edema causes of ICHTN [137].

A CPP driven approach for the management of TBI may prevent secondary injury from hypoperfusion (e.g., cerebral ischemia) and hyperperfusion (e.g., edema), but the accurate estimation of “true CPP” relies on ICP and MAP. For CPP determination, the transducer for measuring blood pressure should be referenced at the level of tragus rather than the heart [130]. However, in many clinical practices blood pressure transducers are referenced at the level of heart, which has significant implications on the determination of CPP [138,139]. There is no standardized calibration site for measurement of MAP for CPP estimation. Moreover, optimal CPP is patient specific, thus there is no set standard for the management of TBI other than adhering to the widely accepted range of CPP. CPP is only a surrogate of cerebral blood flow, which implies that changes in cerebral vascular resistance may not be detected by CPP.

### Advanced ICP Analysis for TBI

Modern computational algorithms are being developed to facilitate an efficient management of TBI. Most algorithms are focused on the derivation of event predictors based on ICP waveform analysis. A Morphological Clustering and Analysis of ICP (MOCAIP) algorithm is described by Hu *et al.* [140], which forecasts elevation in ICP based on prior changes in the morphology of the ICP waveform. The algorithm quantitates the characterization of ICP waveform morphology by extracting 24 morphological metrics, including ICP mean amplitude, time between three pulses (P1, P2, and P3), curvature, slope, and decay time constant. This retrospective study was conducted on data from patients suffering from idiopathic ICHTN, shunt malfunction, adult slit ventricle syndrome, and Chiari malformation. This MOCAIP methodology was able to predict ICP elevation 5 min prior to the onset of the event with >97% specificity and ~80% sensitivity. An advanced form of this algorithm has the potential for forecasting events in TBI patients who are undergoing continuous ICP monitoring. Indices (cerebrovascular pressure–reactivity and cerebrospinal compensatory reserve (RAP)) can be helpful for the interpretation of progressive ICHTN in patients after TBI [116]. ICP analysis can also be utilized to determine the association between patterns of ICHTN and outcome after TBI. An early onset of ICHTN was found to be significantly associated with unfavorable outcome after severe TBI with a high correlation between the duration of ICHTN and mortality [141].

## 6. Conclusions

The advances in the technique of ICP monitoring provide a variety of methods for assessing ICP. These techniques are able to record ICP in real time, at the bedside, and they allow therapeutic interventions by detecting changes in intracranial pathophysiology. ICP is a complex variable, which is used to derive information about CPP, cerebral compensatory mechanisms, and autoregulation. The ability to continuously monitor ICP, analyze ICP waveform, and derive cerebral indices is pivotal in understanding pathology, targeting therapy, and predicting prognosis. The technique most commonly used in clinical practice to monitor ICP involves intraventricular or intraparenchymal catheter system, which is still considered the gold standard for ICP monitoring. Further work is required to confirm the accuracy and applicability of non-invasive methods for ICP measurements. Precise diagnosis and the choice of therapy will require establishing patient specific thresholds for ICP in combination with other physiologic parameters that can optimize its predictive capacity. Despite some controversies about the role of ICP monitoring in TBI, it remains robust and widely applicable as a valuable indicator of TBI severity.
